# Supplementary material for: Concurrent representations of reinstated and transformed memories and their modulation by reward
Source: Imaging Neurosci (Camb). 2025 Feb 18;3:imag_a_00476. doi: 10.1162/imag_a_00476 (PMC12319746; doi:10.1162/imag_a_00476)
Supplement: Supplementary Material [file imag_a_00476-supp.pdf]

Concurrent representations of reinstated and transformed memories and their modulation by reward

- Supplemental information -

Heidrun Schultz[1,2]*, Hanna Stoffregen[1]*, Aroma Dabas[1], María Alcobendas[1,3], and Roland G. Benoit[1,4]

[1]Max Planck Research Group Adaptive Memory, Max Planck Institute for Human Cognitive and Brain Sciences, Leipzig, Germany

[2]Chair of Lifespan Developmental Neuroscience, TUD Dresden University of Technology, Dresden, Germany

[3]Department of Neurology, Charité-Universitätsmedizin Berlin, Berlin, Germany

[4]Department of Psychology and Neuroscience & Institute of Cognitive Science, University of Colorado Boulder, CO, USA

*These authors contributed equally to this work.

1 Encoding-Retrieval Similarity in the anterior vs. posterior hippocampus

Figure S1 depicts the average encoding-retrieval similarity values in all conditions, separately for the anterior and posterior hippocampus.

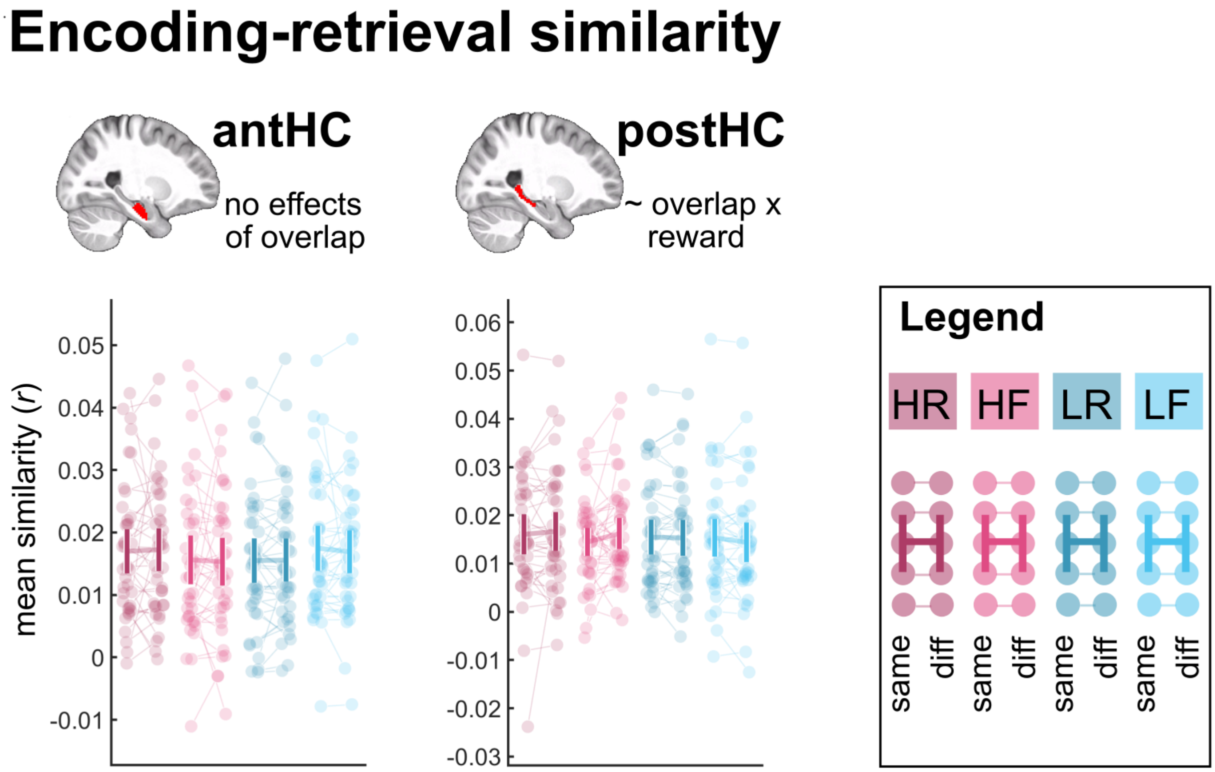

Figure S1. Encoding-retrieval similarity, separately for the anterior vs. posterior hippocampus. Notes refer to effects from three-way repeated-measures ANOVAs with the factors reward, memory, and scene overlap (see main article for details). Error bars indicate 95% confidence interval. Abbreviations: HR: high-reward/remembered; HF: high-reward/forgotten; LR: low-reward/remembered, LF: low-reward/forgotten; same: same-scene similarity, diff: different-scene similarity, antHC: anterior hippocampus, postHC: posterior hippocampus; *r* refers to mean Fisher-z transformed Pearson correlation coefficients. ~ 0.05<*p*<.1.

2 Post-hoc power analysis: Behavioral reward effects

Our incidental encoding task and stimuli are adapted from (Gruber et al., 2016). Gruber et al. found a medium-sized behavioral reward effect on memory ($t_{(18)}$=2.16, resulting in *dz*=2.16/√(19)=0.50)

(Lakens, 2013). At n=40, our study had a power of β=.92 to replicate this effect (assuming α=.05, one-tailed paired t-test in G*Power) (Faul et al., 2009).

3 Univariate analysis: Mesolimbic response to the reward manipulation

Given the non-significant effects of reward on encoding responses and next-day memory in our study, we tested whether our reward manipulation succeeded in engaging the brain's reward system. Reward enhancement of memory has been linked to activity in the mesolimbic system, including the substantia nigra/ventral tegmental area (SN/VTA) and ventral striatum (VS) (Adcock et al., 2006; Wittmann et al., 2005).

To this end, we computed a univariate analysis. We set up first-level GLMs on each participant's encoding data, with categorical regressors on the onsets of the simulation phase (scene + object) and feedback phase of the trials (cf. Figure 1A in the main article), separately for high and low reward. Models were otherwise set up identically to the models underlying our main analyses (see main article). That is, regressors were were convolved with the hemodynamic response function (HRF). Additionally, each model included a set of seven non-convolved noise regressors extracted during preprocessing (see main article), i.e. the six rigid motion regressors (three translations, three rotations) as well as framewise displacement. Functional runs were concatenated, and session constants were included in the models.

As the reward outcomes were 100% predictable based on scene identity, we expect the dopaminergic response to shift to the reward-predicting cue (i.e. the appearance of the scene-object pair) (Shohamy & Adcock, 2010). Hence, we contrasted high vs. low reward conditions during the simulation phase of the trial in two reward-related regions of interest:

1) The substantia nigra/ventral tegmental area (SN/VTA) (Adcock et al., 2006; Haber & Knutson, 2010; Wittmann et al., 2005). To this end, we used subject-specific anatomical ROIs from n=39 subjects from a previous study (Schultz et al., 2022). We normalized these to MNI space, averaged them over subjects, and thresholded the average at >0.5 (i.e., we included voxels with >50% intersubject agreement). We then inverse-normalized the resulting MNI-space ROI to the individual space of each subject of the current study, similar to the mPFC ROI in the main article.

2) The ventral striatum (VS) (Haber & Knutson, 2010). This mask was taken from the Oxford-GSK-Imanova atlas (Tziortzi et al., 2011) and inverse-normalized as above.

We extracted and averaged beta values for the high vs. low reward condition in each participant and ROI, and contrasted them using paired *t*-tests. We found that:

1) Activity in the SN/VTA was significantly larger for high-reward than low-reward trials ($t_{(39)}$=3.298, *p*=.002, see Figure S1).
2) Activity in the VS was numerically larger for high-reward than low-reward trials, albeit the difference was not statistically significant ($t_{(39)}$=1.038, *p*=.306). However, visual inspection revealed an outlier (see Figure S1, red arrow), whose reward effect (high reward minus low reward) was ~4.47 standard deviations below the mean effect. After removing this participant from the analysis, activity in the VS was significantly larger for high-reward than low-reward trials ($t_{(38)}$=2.945, *p*=.006).

Taken together, these results imply that the reward system was engaged by the high reward condition.

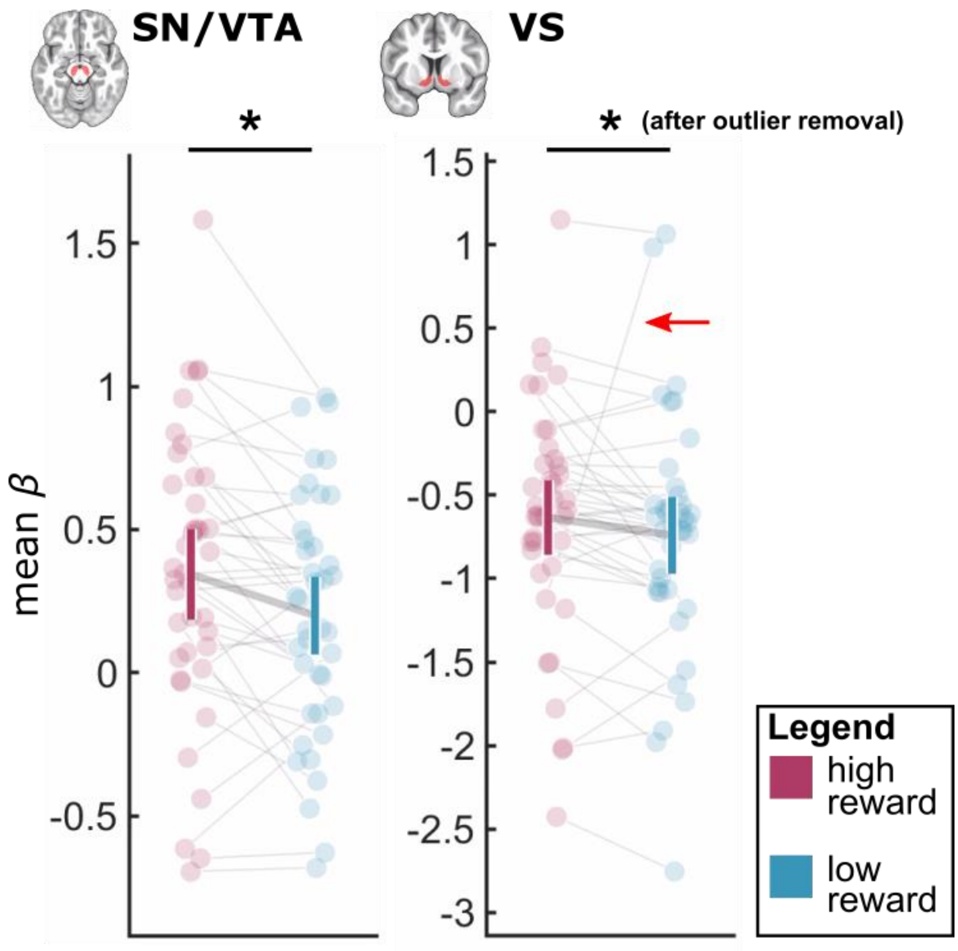

Figure S2. Univariate responses during the incidental encoding task. Error bars indicate 95% confidence interval. *$p<.05$, red arrow: Outlier that was removed for analysis of VS.
